# Limited Impact of Imatinib in a Murine Model of Sclerodermatous Chronic Graft-versus-Host Disease

**DOI:** 10.1371/journal.pone.0167997

**Published:** 2016-12-12

**Authors:** Ludovic Belle, Gilles Fransolet, Joan Somja, Marilène Binsfeld, Philippe Delvenne, Pierre Drion, Muriel Hannon, Yves Beguin, Grégory Ehx, Frédéric Baron

**Affiliations:** 1 Hematology Research Unit, GIGA-I³, University of Liège, Liège, Belgium; 2 Department of Pathology, University of Liège, Liège, Belgium; 3 GIGA-R, University of Liège, Liège, Belgium; 4 Department of Medicine, Division of Hematology, CHU of Liège, Liège, Belgium; University of Kentucky, UNITED STATES

## Abstract

**Background:**

Sclerodermatous chronic Graft-versus-Host Disease (scl-cGVHD) is one of the most severe form of cGVHD. The Platelet-derived Grotwth Factor (PDGF) and the Transforming Growth Factor-β (TGF-β) play a significant role in the fibrosing process occurring in scl-cGVHD. This prompted us to assess the impact of the PDGF-r and c-Abl tyrosine kinase inhibitor imatinib on scl-cGVHD.

**Methods:**

To assess the impact of imatinib on T cell subset proliferation *in vivo*, Balb/cJ recipient mice were lethally (7 Gy) irradiated and then injected with 10x10^6^ bone marrow cells from B10.D2 mice on day 0. Fourteen days later, 70x10^6^ carboxyfluorescein succinimidyl ester (CFSE)-labeled splenocytes from B10.D2 mice were infused and imatinib or sterile water was administered for 5 days. To induce severe scl-cGVHD, Balb/cJ mice were injected i.v. with 10.10^6^ bone marrow cells and 70.10^6^ splenocytes from B10.D2 donor mice after 7 Gy irradiation. Mice were then given sterile water or imatinib from day +7 after transplantation to the end of the experiment (day +52).

**Results:**

Imatinib decreased the proliferation of total T cells (*P* = 0.02), CD8+ T cells (*P* = 0.01), and of regulatory T cells (Tregs) (*P* = 0.02) in the spleen. In the severe scl-cGVHD model, imatinib-treated mice had significantly lower levels of PDGF-r phosphorylation than control mice on day 29 after transplantation (*P* = 0.008). However, scl-cGVHD scores were similar between vehicle- and imatinib-treated mice during the whole experiment, while there was a suggestion for less weight loss in imatinib-treated mice that reached statistical significance at day +52 following transplantation (*P* = 0.02).

**Conclusions:**

Imatinib had a limited impact in murine scl-cGVHD despite significant inhibition of PDGF-r.

## Introduction

Allogeneic hematopoietic stem cell transplantation (allo-HSCT) is the main curative treatment for many hematological malignancies [1]. Its anti-tumor activity relies in large part on immune-mediated graft-versus-tumor effects (GvT effects) [2, 3]. However, donor immune cells contained in the graft can also attack healthy host tissues causing graft-versus-host disease (GVHD) [4–7]. GVHD can be divided into two syndromes, acute GVHD, historically defined as a GVHD reaction occurring within the first 100 days after allo-SCT and chronic GVHD (cGVHD), that generally occurs beyond day 100 [8, 9]. While cGVHD has been associated with graft-versus-tumor effects [3, 10], it is also a major cause of morbidity/mortality in long-term transplant recipients [11].

Sclerodermatous cGVHD (scl-cGVHD) is one of the most severe form of cGVHD and develops in approximately 20% of cGVHD patients [12]. Although scl-cGVHD shares common features with systemic fibrosis, the two syndromes differ both in terms of pathology (scl-cGVHD usually begins in the superficial layer of the skin and then extents to deeper layers of the skin while the opposite is generally true in systemic sclerosis), and in terms of clinical symptoms, with clinical features such as Raynaud’s syndrome, pulmonary hypertension and cardiac dysfunction being frequently observed in patients with systemic sclerosis but infrequently in scl-cGVHD patients [13, 14].

The pathogenesis of cGVHD remains not fully understood. It is generally accepted that donor T cells are largely involved [4]. Specifically, data from murine models of cGVHD suggest that donor T cells involved in cGVHD are mainly CD4^+^ T helper 2 (Th2) cells [15]. These Th2 cells secrete IL-4, IL-5, IL-10, IL-11 and IL-13 that stimulate other cells to release fibrosing factors such as IL-13, PDGF and TGF-β.These ones then induce fibrosis in the skin and other affected organs. Histocompatibility antigenic disparities between donor and recipient are also a risk factor for cGVHD (although to a lesser extent than for acute GVHD [16]), suggesting that cGVHD manisfestations are due to recognition of allogeneic antigens, such as major or minor histocompatibility antigens by donor T cell. Host thymus integrity could also play a role, as suggested by the lower incidence of chronic GVHD in younger recipients [16], although some studies failed to observe an association between thymic function and subsequent occurrence of cGVHD [17, 18]. Finally, emerging data have also demonstrated an important role for B cells in cGVHD pathogenesis [19–21].

Imatinib (Glivec^®^; Novartis Pharmaceuticals) is a tyrosine kinase inhibitor developed as a competitive inhibitor of ATP for binding to BCR-ABL inducing apoptosis of BCR-ABL dependent leukemic cells [22]. However, imatinib is not specific towards BCR-ABL and also targets other tyrosine kinases such as the stem cell factor c-kit, c-Abl (involved in transforming growth factor (TGF)-β signaling pathway), and platelet-derived growth factor receptor (PDGF-r) [22]. Given that the TGF-β and PDGF signaling pathways are largely involved in the fibrogenesis process in scl-cGVHD [15, 23], and given the ability of imatinib to inhibit T-cell proliferation *in vitro* [24], some clinical studies have assessed the impact of imatinib in patients with steroid-refractory cGVHD [25–29]. Unfortunately, these studies yielded conflicting results underlying the importance of re-assessing the impact of imatinib in scl-cGVHD in pre-clinical models. Here we investigated the impact of imatinib on scl-cGVHD in a classical scl-cGVHD murine model (B10.D2 (H-2^d^) → BALB/cJ (H-2^d^)) [15, 30].

## Material and Methods

### Mice and drugs

Twelve to 14 week-old B10.D2 (H-2^d^, Jackson Laboratories, Bar Harbor, USA) and Balb/cJ (H-2^d^, Jackson Laboratories) mice were used as donors and recipients, respectively, in a MHC-matched minor antigens disparate scl-cGvHD model [30, 31]. All mice were maintained in top-filtered cages in a standard animal facility and provided with sterilized food. Sterilized water supplemented with Baytril^®^ 1% (Bayer HealthCare, Diegem, Belgium) was given from 3 days before transplantation until the end of the experiment (day +52). Water was changed every 2–3 days. All animal experiments were approved by the animal ethic committee of the University of Liege (local file number: 1438).

Imatinib (Glivec^®^) was kindly provided by Novartis Pharmaceuticals (Basel, Switzerland). Imatinib was dissolved in sterile water and given by oral gavage at a dose of 150 mg/kg/day (50 mg/kg every morning + 100 mg/kg every evening).

### Cell preparation for bone marrow transplantation

Spleen, femurs and tibias from B10.D2 mice were collected in sterile RPMI + FBS 10% + Penicilline/Streptomycine (P/S) 1%. After red blood cell lysis (using RBC lysis buffer; eBioscience, San Diego, USA), suspensions were passed through a 70 μm nylon filter and washed with PBS + FBS 3% + P/S 1%. Cells were then resuspended and mixed in pure PBS at a concentration of 70x10^6^ spleen cells and 10x10^6^ bone marrow cells/200 μL (severe scl-cGVHD model) or 2x10^6^ spleen cells and 1x10^6^ bone marrow cells/200 μL (moderate scl-cGVHD model).

### Bone marrow transplantation and scl-cGVHD

Balb/cJ recipient mice were transplanted as previously described [31]. Briefly, recipient Balb/cJ mice (H-2^d^) were lethally irradiated with 7 Gy total body irradiation using a ^137^Cs irradiator (GammaCell 40, Nordion, Ontario, Canada). Six hours later, recipients were injected with 200 μL of the prepared cell suspension (2x10^6^ spleen cells + 1x10^6^ bone marrow cells for the moderate scl-cGVHD model [32] or 70x10^6^ spleen cells + 10x10^6^ bone marrow cells for the severe scl-cGVHD model [31, 33]) from B10.D2 (H-2^d^) mice. Recipient mice were monitored daily, while recipient body weights and GVHD scores (see next section) were recorded every 3 days. Mice were observed for a maximum of 52 days after transplantation. Imatinib treatment was started on day +7 post-transplant and was prepared in sterile water. A dose of 150 mg/kg/day (50 mg/kg every morning + 100 mg/kg every evening, as previously reported [34]) was given orally, while control mice received the same volume of sterile water. This high dose of imatinib has been used in several previous *in vivo* studies [32, 35, 36]. At the end of the experiment (day +52), mice were sacrificed and splenocytes and bone marrow cells were collected in sterile RPMI + FBS 10% + P/S 1% while blood was collected in Microtainer^®^ K2E tubes (BD Biosciences, San Diego, California, USA).

### Assessment of scl-cGVHD

Scl-cGvHD severity was assessed using a clinical scoring system as previously reported [31, 33]. Briefly, ear-tagged animals were individually scored 3 times/week for five parameters: weight loss (grade 1, loss between 10–20%; grade 2, loss > 20%), posture (1, kyphosis only at rest; 2, severe kyphosis when the animal moves), activity (1, moderate activity impairment; 2, no move unless stimulated), skin (1, erythema or scaling tail; 2, lesions on the body surface), and hair loss (1, loss > 1 cm^2^; 2 loss > 2 cm^2^). The clinical score was generated by summation of the five criteria scores (0 to 10/10). Animals reaching a score of 8/10 were sacrificed to avoid unnecessary pain according to our local ethic committee. Final scores for dead animals reaching the ethical limit score were carried forward for the remaining time points.

### Skin biopsies and immunohistochemistry

On day 29 after transplantation, mice were anesthetized with an i.p. injection of a solution of xylasin/ketamin (10 mg/kg and 75 mg/kg, respectively, given in i.p.). Skin tissue samples of 0.5 cm^2^ from the upper back were collected and directly fixed in paraformaldehyde 10% before being paraffin-embedded and sectioned at 4–5 μm thickness. Parrafin-embedded sections were deparaffinized in xylene and rehydrated through a graded ethanol series. Antigen was retrieved with citrate buffer combined with heating in a microwave oven. Slides were peroxidase blocked with a commercial protein block serum-free solution (Dako, Glostrup, Danemark) for 10 minutes and were then incubated with one of the following antibodies at 4°C overnight: polyclonal rabbit anti-mouse phospho–c-Abl or polyclonal rabbit anti-mouse phospho-PDGFR (Abcam PLC, Cambridge, United Kingdom) and detected with the Envision system (Dako) for 30 minutes. Colorimetric detection was completed with diaminobenzidine (Dako) and slides were then counterstained with hematoxylin. Phospho-c-Abl and phospho-PDGF-r was evaluated in the dermis by a semi-quantitative score of the intensity and extent of staining according to an arbitrary scale

### *In vivo* proliferation experiment

Balb/cJ recipient mice were lethally (7 Gy Total body irradiation) irradiated and then injected with 10x10^6^ B10.D2 bone marrow cells on day 0. Fourteen days later, spleens from B10.D2 donor mice were harvested and cells were isolated as previously described. Splenocytes were labeled with carboxyfluorescein succinimidyl ester (CFSE, Invitrogen, Carlsbad, CA, USA) according to the manufacturer’s instructions. Cells were then resuspended in PBS at a concentration of 70x10^6^ cells/200 μL. Recipient mice were injected i.v. with 70x10^6^ cells and received imatinib or sterile water treatment for 5 days as described above. Mice were then sacrificed and blood, lymph nodes and spleen were collected. Harvested cells were then stained for CD3, CD4, CD8 and FoxP3 expression.

### Flow cytometry

The reconstitution of the different T-cell sub-populations was evaluated by flow cytometry at the indicated time-points. Specifically, blood from tail vein was collected in a BD Microtainer^®^ K2E Tube (BD Biosciences, San Diego, USA). Blood volumes were measured and transferred into a FACS tube. Blood cells were washed with PBS + FBS 3% + P/S 1% (staining buffer) and supernatants were then removed. Biotinilated antibody was added and incubated with cells for 30 minutes in the dark at 4°C. Cells were washed twice with staining buffer. Extracellular antibodies, including the streptavidin antibody, were added as a mix directly in tubes and incubated for 30 minutes at 4°C in the dark. Cells were washed twice with staining buffer. Cells were then fixed and permeabilized using the FoxP3 staining buffer set (eBioscience, San Diego, CA, USA) according to the manufacturer’s instructions. Cells were then incubated for 30 minutes at 4°C in the dark with intracellular antibodies and finally washed twice and resuspended with pure PBS in BD Trucount Tubes^®^ to determine the absolute number of each lymphocyte population per μL of blood.

The following antibodies were used: anti-mouse CD3 V500 (clone: 500A2, BD Biosciences), CD4 eFluor 450 (clone: RM4-5, eBiosciences), CD8 Pe-Cy7 (clone: 53–6.7, eBiosciences), CD4 FITC (clone: GK 1.5, eBioscience), CD8 APC-Cy7 (clone: 53–6.7, eBioscience), CD62L Pe-Cy7 (clone: MEL-14, eBioscience), CD44 PerCP-Cy5.5 (clone: IM7, eBioscience), CD44 FITC (Clone: IM7, eBioscience) and anti-mouse FoxP3 APC (clone: FJK-16s, eBioscience) for T-cell subpoplations; and anti-human Ki-67 PE or PerCP-Cy5.5 (Clone: B56, BD Biosciences) was used to assay T cells in proliferation.

Data were acquired on a FACS Canto II (Becton Dickinson) and were analyzed with FlowJo 10.0.7 (Treestar Inc., San Carlos, CA).

### Statistics

Statistical analyses were performed with the Graphpad® Prism 5.00 software (La Jolla, CA, US). The Mann-Whitney U test was used to compare GVHD or histological scores and flow cytometry data in the different experimental groups. Results were expressed as median ± interquartile range or mean ± SEM with P-values considered as significant at ≤ 0.05. All P-values were 2-sided.

## Results

### Imatinib decreases *in vivo* proliferation of T-cell subsets

**Fig 1:**
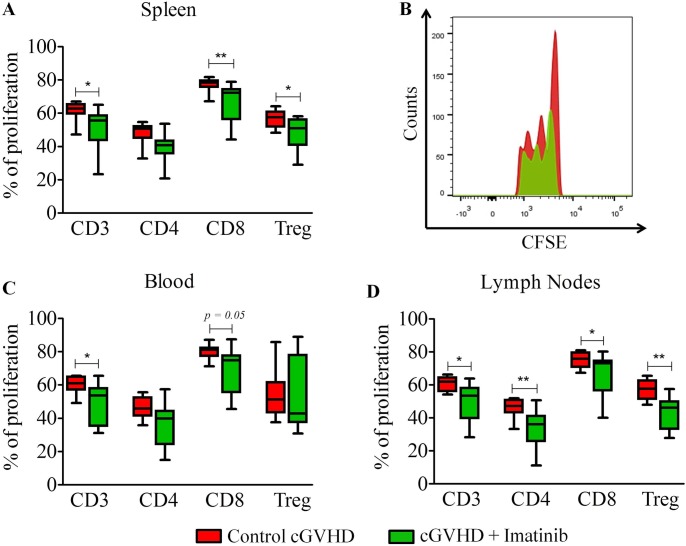
Imatinib decreases *in vivo* proliferation of T-cells. Balb/cJ mice were lethally irradiated and then injected i.v. with 10x10^6^ bone marrow cells from B10.D2 mice on day 0 and 70x10^6^ CFSE-labelled splenocytes on day +14. Mice were then treated with sterile water (n = 10) or imatinib (n = 12) by oral gavage at the dose of 150 mg/kg/day (50 mg in the morning and 100 mg in the evening) directly after splenocytes injection until sacrifice 5 days later. Proliferation was then assessed among total CD3+ T cells, CD4+ T cells, CD8+ T cells and regulatory T cells (Tregs) from imatinib-treated or control mice in the spleen **(A)**, blood **(C)** and lymph nodes **(D)**. **(B)** Representative histogram showing CFSE dilution of total CD3^+^ T-cells in the spleen. Results are expressed as median, 25^th^ and 75^th^ percentiles of the distribution (boxes) and whiskers extending to 5^th^ and 95^th^ percentiles. *P<0.05, **P<0.01. Mann-Whitney test.

Given the primordial role of donor T cells in the pathogenesis of cGVHD, we first assessed the impact of imatinib on T-cell proliferation *in vivo*. Specifically, after 7 Gy TBI, Balb/cJ mice were transplanted with 10x10^6^ bone marrow cells from B10.D2 mice on day 0, followed by the injection of 70x10^6^ CFSE-labeled spleen cells on day + 14. Treatment with sterile water or imatinib was started immediately after splenocyte injection. Mice were sacrificed 5 days later and spleen, lymph nodes, blood and bone marrow were collected and stained for flow cytometry experiments. Imatinib decreased the proliferation of total T cells (defined as CD3^+^ cells) in the spleen (*P* = 0.018), the blood (*P* = 0.0271), and the lymph nodes (*P* = 0.015), the proliferation of CD4^+^ T cells in the lymph nodes (*P* = 0.005), the proliferation of CD8^+^ T cells in the spleen (*P* = 0.007), the blood (*P* = 0.05), and the lymph nodes (*P* = 0.044), and the proliferation of Tregs in the spleen (*P* = 0.023) and the lymph nodes (*P* = 0.002) (Fig 1).

### Imatinib does not affect cGVHD severity

**Fig 2:**
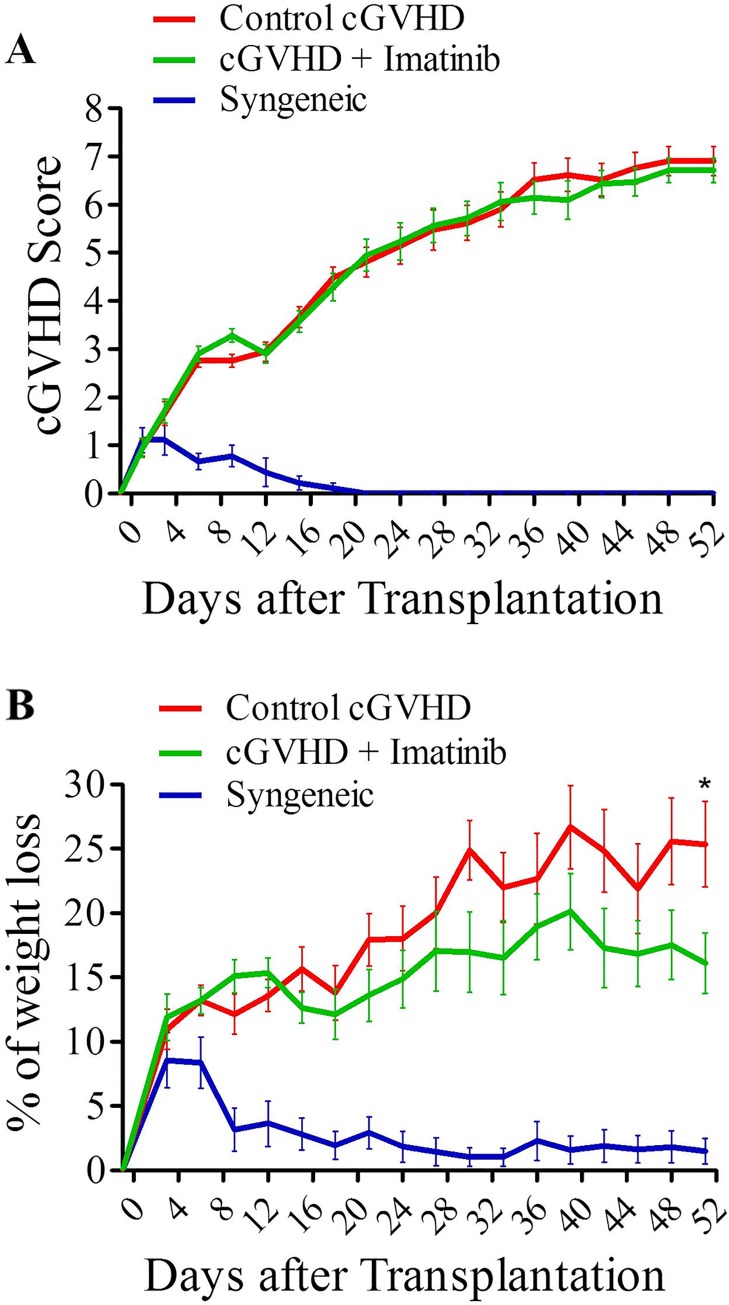
Imatinib does not affect cGVHD severity. Balb/cJ mice were injected i.v. with 10x10^6^ bone marrow cells and 70x10^6^ splenocytes from B10.D2 donor mice after lethal irradiation at 7 Gy. Mice were then given sterile water (n = 21) or imatinib (n = 21) by gavage at the dose of 150 mg/kg/day (50 mg in the morning and 100 mg in the evening) from day +7 post-transplant to the end of the experiment (day +52). **(A)** Pooled GVHD scoring of three independent groups of mice given or not imatinib, showing no impact of the treatment on cGVHD scores. (B) Evolution of mice weight loss during the experiment showing slightly lower weight loss for imatinib-treated mice reaching statistical significance on day +52. Results are expressed with mean with SEM. *P<0.05

Fig 2A shows the combined results of 3 consecutive experiments in the severe scl-cGVHD model. Imatinib did not impact cGVHD severity since a comparable evolution of cGVHD scores was observed in the two groups of mice although there was a suggestion for less weight loss in imatinib-treated mice that reached statistical significance at day +52 following transplantation (*P* = 0.0226) (Fig 2B). Moreover, imatinib did not reduce scl-cGVHD scores in the moderate model of scl-cGVHD (S1 Fig).

### Imatinib decreases phosphorylation of PDGF-r and C-Abl

**Fig 3:**
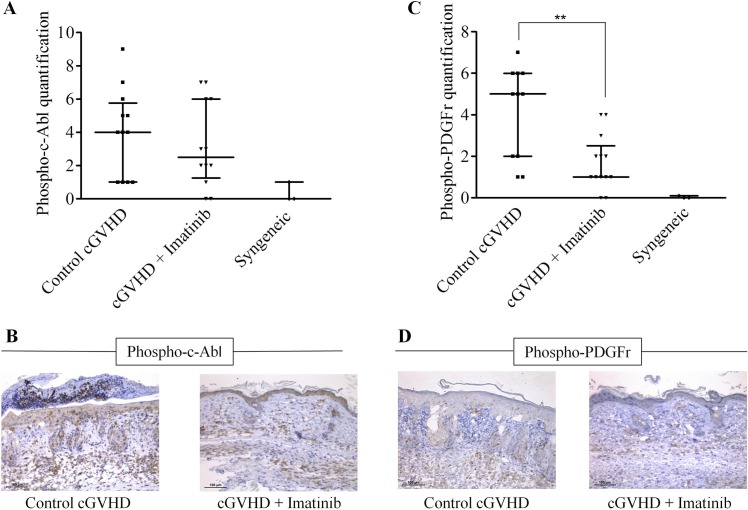
Imatinib decreases phosphorylation of PDGF-r and c-Abl. Skin samples from the upper back of each mice were harvested on day +29 post-transplant and directly fixed in formol 10% before being paraffin-embedded. Skin sections were then stained using anti-phospho-PDGFR and anti-phospho-c-Abl to quantify phosphorylation levels of these receptors in the dermis. Samples were quantified by multiplying the staining intensity (scored 0 to 3) by the extent of stained area (scored 0 to 3). **(A-B)** Immuno-histological evaluation of the phosphorylation level of c-Abl (control: n = 12, imatinib: n = 12, syngeneic: n = 3). **(C-D)** immune-histological evaluation of the phosphorylation level of the PDGF-receptor (control: n = 11, imatinib: n = 13, syngeneic: n = 3).*P<0.05. Results are expressed in median with interquatile range, Mann-Whitney test.

We next assessed on skin biopsy harvested on day +29 whether PDGF-r and c-Abl signaling pathways were impacted by imatinib. Indeed, it has been previously reported that prevention of fibrosis by tyrosine kinase inhibitors could be attributed at least in part to a direct inhibition of c-Abl and PDGF-r signaling [32]. As shown in Fig 3, there were comparable levels of phosphorylated c-Abl on day 29 in imatinib-treated and control mice (*P* = 0.6), while imatinib-treated mice had significantly lower levels of PDGF-r phosphorylation than control ones (*P* = 0.0079) (Fig 3). These data demonstrate that imatinib inhibits PDGF-r signaling.

### Imatinib does not affect T-cell counts on day +21

We next assessed the impact of imatinib on T-cell subsets in scl-cGVHD mice 14 days after treatment initiation (D+21 post-transplantation).

#### - Comparison between severe scl-cGVHD and syngeneic transplants

**Fig 4:**
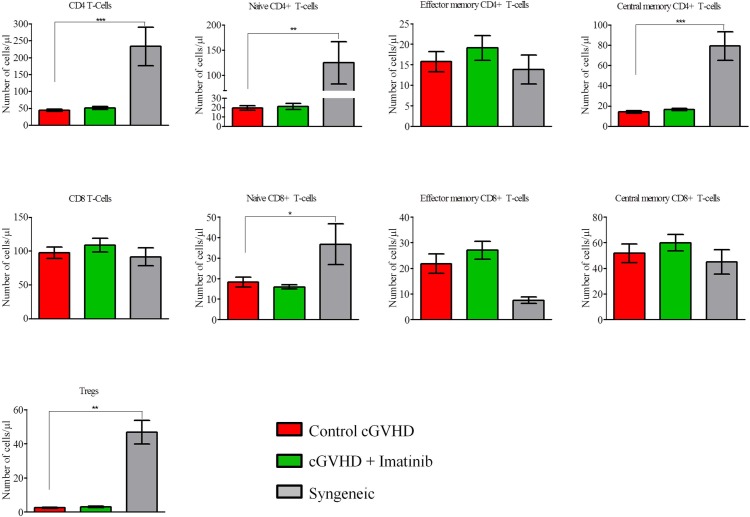
Imatinib does not affect T-cell (subset) counts at day +21. Blood samples from transplanted mice were collected at day +21 post-transplant to assess absolute numbers of T-cell subpopulations. Results indicate that imatinib does not affect absolute numbers of CD4^+^ T-cells, CD8^+^ T-cells or Tregs. *P<0.05; **P<0.01; ***P<0.001. Control: n = 12, imatinib: n = 12, syngeneic: n = 6. Results are expressed in mean with SEM, Mann-Whitney test.

Fig 4 shows that in comparison with severe scl-cGVHD control mice, animals given syngeneic grafts had higher absolute counts of CD4^+^ T cells (*P* = 0.0009), naïve CD4^+^ T cells (*P* = 0.0032), naïve CD8^+^ T cells (*P* = 0.0333), and Tregs (*P* = 0.0019) in the peripheral blood.

**Fig 5:**
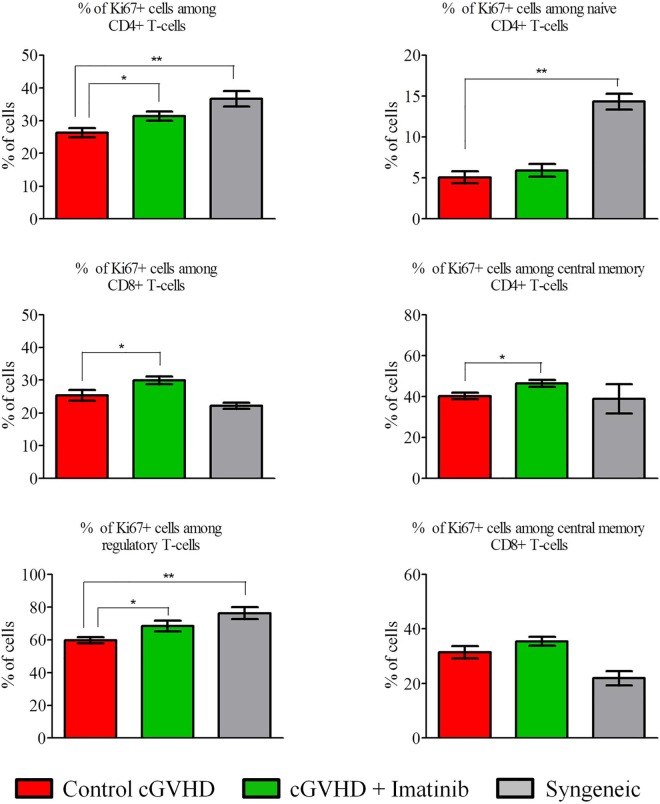
Imatinib increases T-cell proliferation at day +21. Blood samples were collected at day +21 post-transplant to assess T-cell (subpopulation) proliferation (measured by KI67 expression). The results indicate that the expression of Ki67 by CD4+ T cells, CD8+ T cells and Tregs was higher in imatinib-treated mice (n = 12) than in controls (n = 12), while no difference was observed for the other T-cell subsets. *P<0.05; **P<0.01. Syngeneic mice: n = 5. Results are expressed in mean with SEM, Mann-Whitney test.

We also compared the proliferation of the different T-cell subsets on day 21 by measuring their expression of KI67. As shown in Fig 5, compared to severe scl-cGVHD control mice, mice given syngeneic grafts had higher proliferation of CD4^+^ T cells (*P* = 0.0072), naïve CD4^+^ T cells (*P* = 0.0019) and Tregs (*P* = 0.0061) but lower proliferation of CD8^+^ T cells (*P* = NS).

#### - Comparison between control and imatinib-treated cGVHD mice

As shown in Fig 4, absolute counts of the various T-cell subsets were comparable in imatinib-treated and control mice in the severe scl-cGVHD model. In the moderate scl-cGVHD model, in comparison to control mice, mice given imatinib had slightly lower counts of total (*P* = 0.0101) and of effector memory (*P* = 0.048) CD4^+^ T cells, but higher counts of central memory CD8^+^ T cells (*P* = 0.0333) (S2 Fig).

Finally, compared to control mice, imatinib-treated mice displayed higher proliferation of CD4^+^ T cells (*P* = 0.0464), CD8^+^ T cells (*P* = 0.0496), and Tregs (*P* = 0.035) in the severe scl-cGVHD model (Fig 5). This was not observed in the moderate scl-cGVHD model (S3 Fig).

### Imatinib does not affect T-cell counts on day +35

As for day +21, we also assessed the impact of imatinib on T-cell subsets in blood and on their proliferation on day +35 after transplantation (28 days after starting treatments).

#### - Comparison between severe scl-cGVHD and syngeneic transplants

**Fig 6:**
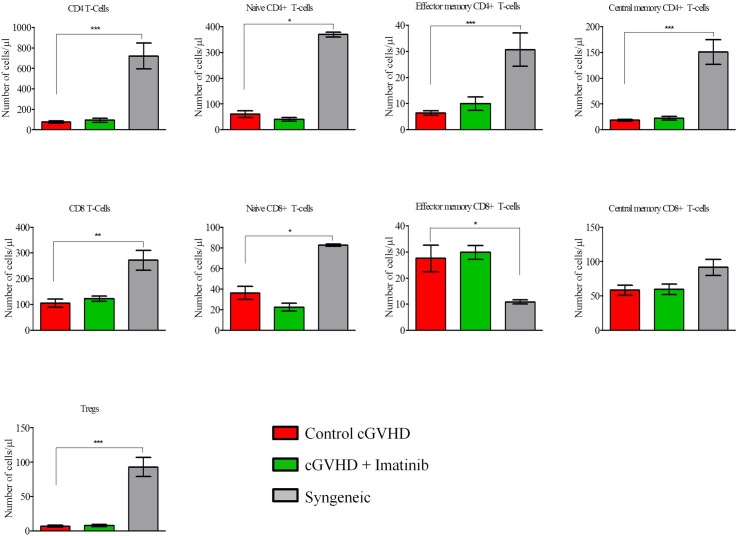
Imatinib does not affect T-cell (subset) counts at day +35. Blood samples were collected to at day +35 post-transplant to assess the impact of the treatment on absolute number of T-cell subpopulations. Imatinib treatment did not affect the counts of T-cell subpopulations. Control: n = 9, imatinib: n = 10, syngeneic: n = 6. *P<0.05; **P<0.01; ***P<0.001. Results are expressed in mean with SEM, Mann-Whitney test.

Fig 6 shows that, compared to severe scl-cGVHD control mice, mice receiving syngeneic grafts had higher absolute counts of all T-cell subsets (P values ranged from 0.0004 to 0.0167) except for effector memory CD8^+^ T-cells that, on the contrary, were higher in severe scl-cGVHD than in syngeneic mice (*P* = 0.0167) and counts of central memory CD8^+^ T cells were comparable in the two groups.

**Fig 7:**
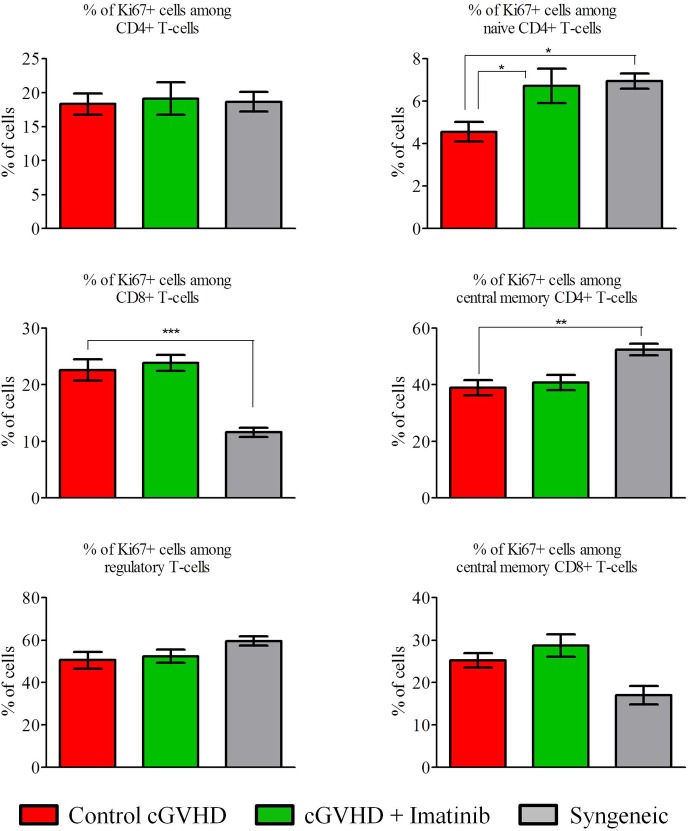
Imatinib does not affect T-cell proliferation at day +35. Blood samples were collected at day +35 post-transplant to assess T-cell proliferation (measured by KI67 expression). Proliferation of naive CD4+ T cells was significantly higher in imatinib- (n = 10) than in water-treated mice (n = 8). Further, compared to scl-cGVHD mice, syngeneic mice (n = 6) had a lower proliferation of CD8+ T cells but a higher proliferation of naive and central memory CD4+ T cells. *P<0.05; **P<0.01; ***P<0.001. Results are expressed in mean with SEM, Mann-Whitney test.

Regarding analyses of proliferation assessed by the expression of KI67, proliferation of CD8^+^ T-cells was lower in syngeneic mice than in severe scl-cGVHD control mice (*P* = 0.0007) while the opposite was observed for naïve (*P* = 0.033) and central memory (*P* = 0.003) CD4^+^ T cells (Fig 7).

#### - Comparison between control and imatinib-treated cGVHD mice

As observed on day +21, absolute counts of T-cell subsets were also comparable whether animals received or not imatinib on day +35 in the severe scl-cGVHD model (Fig 6). In the moderate scl-cGVHD model, in comparison to control mice, mice given imatinib had slightly lower counts of total (*P* = 0.008) and naive (*P* = 0.03) CD4^+^ T cells, and of total (*P* = 0.03) and naive (*P* = 0.008) CD8^+^ T cells (S4 Fig).

Interestingly, proliferation of naive CD4^+^ T cells was higher in imatinib-treated than in control mice both in the severe scl-cGVHD (*P* = 0.03) (Fig 7) and in the moderate scl-cGVHD (*P* = 0.016) model (S5 Fig).

## Discussion

PDGF and TGF-β cell signaling pathways play a significant role in the fibrosing process occurring in scl-cGVHD. Imatinib, a well-demonstrated inhibitor of PDGF and TGF-β signaling pathways (by inhibiting PDGF-r and c-Abl tyrosine kinases), has been studied in patients with scl-cGVHD [25–29]. While some reports suggested a beneficial impact of imatinib in that setting [25–27, 29], this was not confirmed in other clinical studies [28]. This prompted us to investigate the impact of imatinib in a well-characterized murine model of scl-cGVHD in which PDGF-r, c-Abl and TGF-β have pivotal function in scl-cGVHD pathogenesis. Several observations were made.

First, *in vivo* administration of imatinib in the severe scl-cGVHD model was able to inhibit its targets. Indeed, phosphorylation levels of PDGF-r were significantly decreased in imatinib-treated compared to control mice, while perhaps a similar trend was observed for c-Abl. This is in concordance with the observations reported by Zerr *et al*. in a similar murine model of scl-cGVHD than the moderate scl-cGVHD model used in the current study [32].

Secondly, imatinib administration failed to significantly ameliorate severe scl-cGVHD, although there was a trend for less weight losses in imatinib-treated mice. This is in discordance to what has been reported by Zerr *et al*. who observed that imatinib significantly decreased scl-cGVHD scores [32]. The reason for this apparent discrepancy is unclear and is not related to the lower numbers of splenocytes injected (35 times less) by Zerr *et al*. since in our hands, imatinib administration did not decrease scl-cGCHD either when assessed in the moderate scl-cGVHD model (in which similar number of donor cells were infused than in the study by Zerr *et al*.). Further, the total dose of imatinib administered was similar in both studies (150 mg/kg), although it was administered once daily in the Zerr *et al*. study and twice daily in the current study. It has been previously reported that a twice-daily schedule of administration of imatinib was more efficient than a one daily schedule [35]. Taken together our data suggest that a partial inhibition of PDGF-r is insufficient to significantly ameliorate scl-cGVHD. Based on this hypothesis, a more potent PDGF-r and c-abl blockade by combining imatinib with nilotinib could be assessed in this mouse model [37]. On the other hand, a possible negative impact of imatinib on scl-cGVHD is its ability to decrease Treg [38]. Indeed, previous studies have demonstrated that imatinib impaired Treg proliferation and function on one hand [38, 39], and, on the other hand, potentiated antitumor T cell responses in an gastrointestinal stromal tumor model through CD8^+^ T cell activation inducing Treg apoptosis within the tumor by depleting indoleamine 2,3-dioxygenase [40]. In agreement with these data, we demonstrated lower Treg proliferation in the spleen and the lymph nodes by imatinib. Previous studies have demonstrated the ability of Tregs to prevent acute [41], xenogeneic [42, 43], and chronic [40, 44] GVHD, while we have recently observed that the hypomethylating agent azacytidine was able to significantly ameliorate severe scl-cGVHD at least in part by promoting Treg [31]. These observations might justify further studies in murine models of GVHD combining imatinib and azacytidine for severe scl-CGVHD prevention.

Thirdly, compared to mice transplanted with syngeneic grafts, we observed that allogeneic scl-cGVHD mice had higher counts of effector memory CD8^+^ T cells but significantly lower counts of all other T-cell subsets. These data suggest that the scl-cGVHD process induces the expansion of effector memory CD8+ T cells while impacting immune reconstitution probably in part by decreasing thymic neo-generation of T cells and increasing T-cell apoptosis as previously reported [45, 46]. Further, these data are consistent with recent observations by Binsfeld *et al*. in a murine model of graft-versus-myeloma effect consisting in the transplantation of BALB/cJ mice, previously injected with luciferase-transfected MOPC315.BM myeloma cells, with bone marrow cells and splenocytes from B10.D2 mice [33].

Fourthly, we observed that imatinib had no impact on T cell reconstitution in severe scl-cGVHD mice, while it slightly impaired it in the moderate scl-cGVHD model. Previous studies have evidenced the *in vitro* inhibitory effects of imatinib on T-cell activation and T-cell proliferation (through direct inhibition of LCK by imatinib) [24, 47]. In accordance with these *in vitro* published data, we observed that imatinib slightly decreased T-cell proliferation (including Treg proliferation) *in vivo* in a lymphopenic host.

In conclusion, imatinib failed to significantly alleviate scl-cGVHD in a severe murine model of scl-cGVHD despite it partially inhibited PDGF-r.

## Supporting Information

S1 Fig Imatinib does not affect cGVHD severity in a moderate model of scl- cGVHD Balb/cJ mice were injected i.v. with 1x10^6^ bone marrow cells and 2x10^6^ splenocytes from B10.D2 donor mice after lethal irradiation at 7 Gy. Mice were then given sterile water (n = 7) or imatinib (n = 6) by oral gavage at the dose of 150 mg/kg/day (50 mg in the morning and 100 mg in the evening) from day +7 post-transplant to the end of the experiment (day +52). Results are expressed in mean with SEM.
(TIF)

S2 Fig Imatinib decreases CD4^+^ T-cell counts but increases central memory CD8^+^ T cell counts at day +21 in a moderate model of scl-cGVHD. Blood samples from transplanted mice were collected at day +21 post-transplant to assess absolute numbers of T-cell subpopulations. Results indicate that, in a moderate model of scl-cGVHD, imatinib does not affect absolute numbers of T cells except for total as well as effector memory CD4^+^ T-cells that were decreased with imatinib and central memory CD8^+^ T cells that were increased with imatinib. *P<0.05; Control: n = 7, imatinib: n = 5. Results are expressed in mean with SEM, Mann-Whitney test.
(TIF)

S3 Fig Imatinib does not affect T-cell proliferation at day +21 in a moderate model of scl-cGVHD. Blood samples were collected at day +21 post-transplant to assess T-cell (subpopulation) proliferation. The results indicate that the expression of KI67 by CD4^+^ T cells, CD8+ T cells and Tregs was similar in imatinib-treated mice (n = 5) than in controls (n = 7). Results are expressed in mean with SEM, Mann-Whitney test
(TIF)

S4 Fig Imatinib decreases CD4 and CD8 T-cell counts at day +35 in a moderate model of scl-cGVHD Blood samples from transplanted mice were collected at day +35 post-transplant to assess absolute numbers of T-cell subpopulations. Results indicate that, in a moderate model of scl-cGVHD, imatinib decreased absolute numbers of total and naïve CD4^+^ and CD8^+^ T-cells. *P<0.05; **P<0.001. Control: n = 7, imatinib: n = 5. Results are expressed in mean with SEM, Mann-Whitney test.
(TIF)

S5 Fig Imatinib increases CD4^+^ T-cells proliferation at day +35 in a moderate model of scl-cGVHD. Blood samples were collected at day +35 post-transplant to assess T-cell (subpopulation) proliferation. The results indicate that the expression of KI67 by total and naïve CD4^+^ T cells was higher in imatinib-treated mice (n = 5) than in controls (n = 7). *P<0.05. Results are expressed in mean with SEM, Mann-Whitney test.
(TIF)
