# Supplementary figures and images for: Limited Impact of Imatinib in a Murine Model of Sclerodermatous Chronic Graft-versus-Host Disease
Source: PLoS One. 2016 Dec 12;11(12):e0167997. doi: 10.1371/journal.pone.0167997 (PMC5152855; doi:10.1371/journal.pone.0167997)

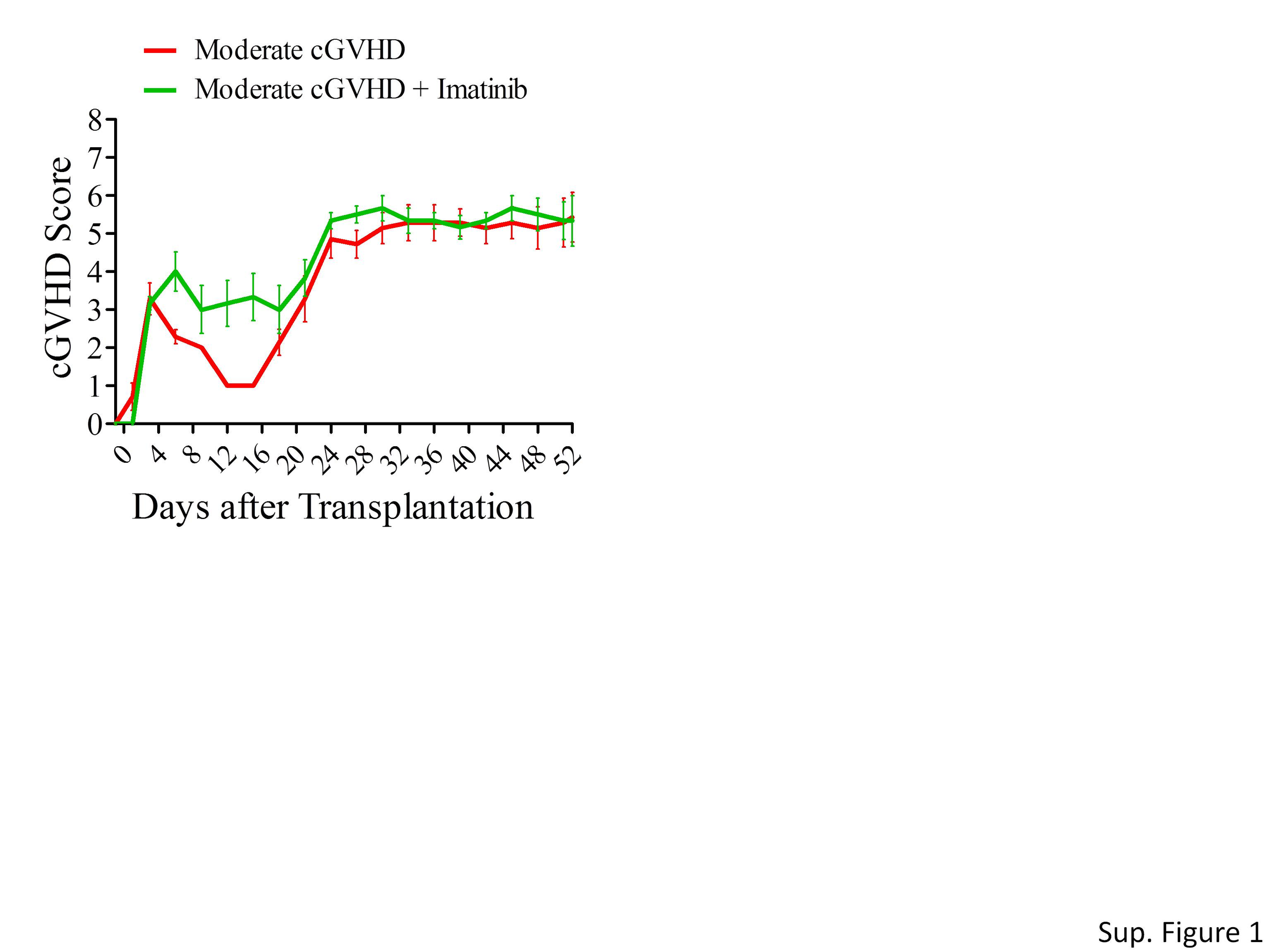

Supplement: S1 Fig — Mice were then given sterile water (n = 7) or imatinib (n = 6) by oral gavage at the dose of 150 mg/kg/day (50 mg in the morning and 100 mg in the evening) from day +7 post-transplant to the end of the experiment (day +52). Results are expressed in mean with SEM. (TIF) [file pone.0167997.s001.tif]

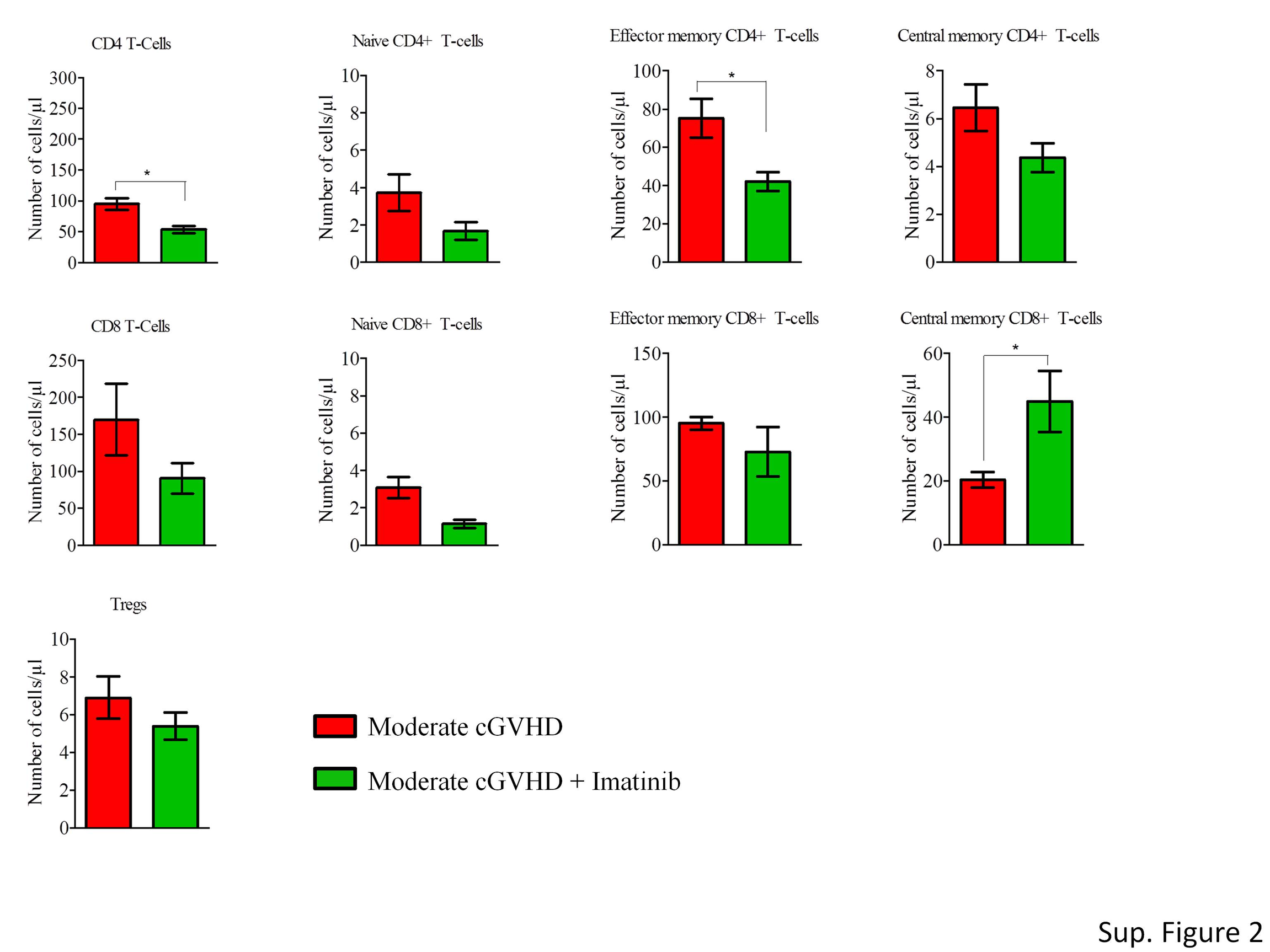

Supplement: S2 Fig — Blood samples from transplanted mice were collected at day +21 post-transplant to assess absolute numbers of T-cell subpopulations. Results indicate that, in a moderate model of scl-cGVHD, imatinib does not affect absolute numbers of T cells except for total as well as effector memory CD4+ T-cells that were decreased with imatinib and central memory CD8+ T cells that were increased with imatinib. *P<0.05; Control: n = 7, imatinib: n = 5. Results are expressed in mean with SEM, Mann-Whitney test. (TIF) [file pone.0167997.s002.tif]

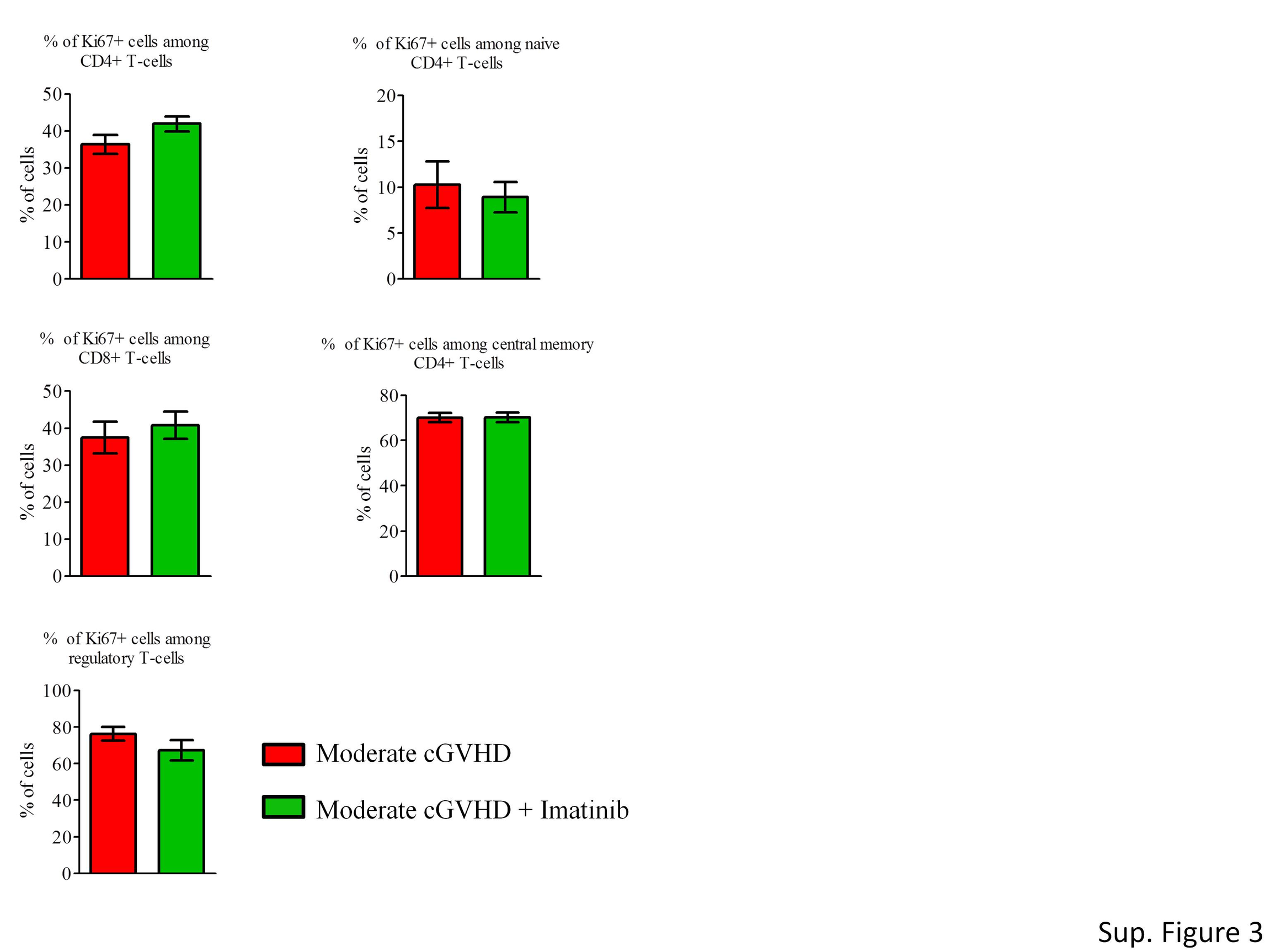

Supplement: S3 Fig — Blood samples were collected at day +21 post-transplant to assess T-cell (subpopulation) proliferation. The results indicate that the expression of KI67 by CD4+ T cells, CD8+ T cells and Tregs was similar in imatinib-treated mice (n = 5) than in controls (n = 7). Results are expressed in mean with SEM, Mann-Whitney test (TIF) [file pone.0167997.s003.tif]

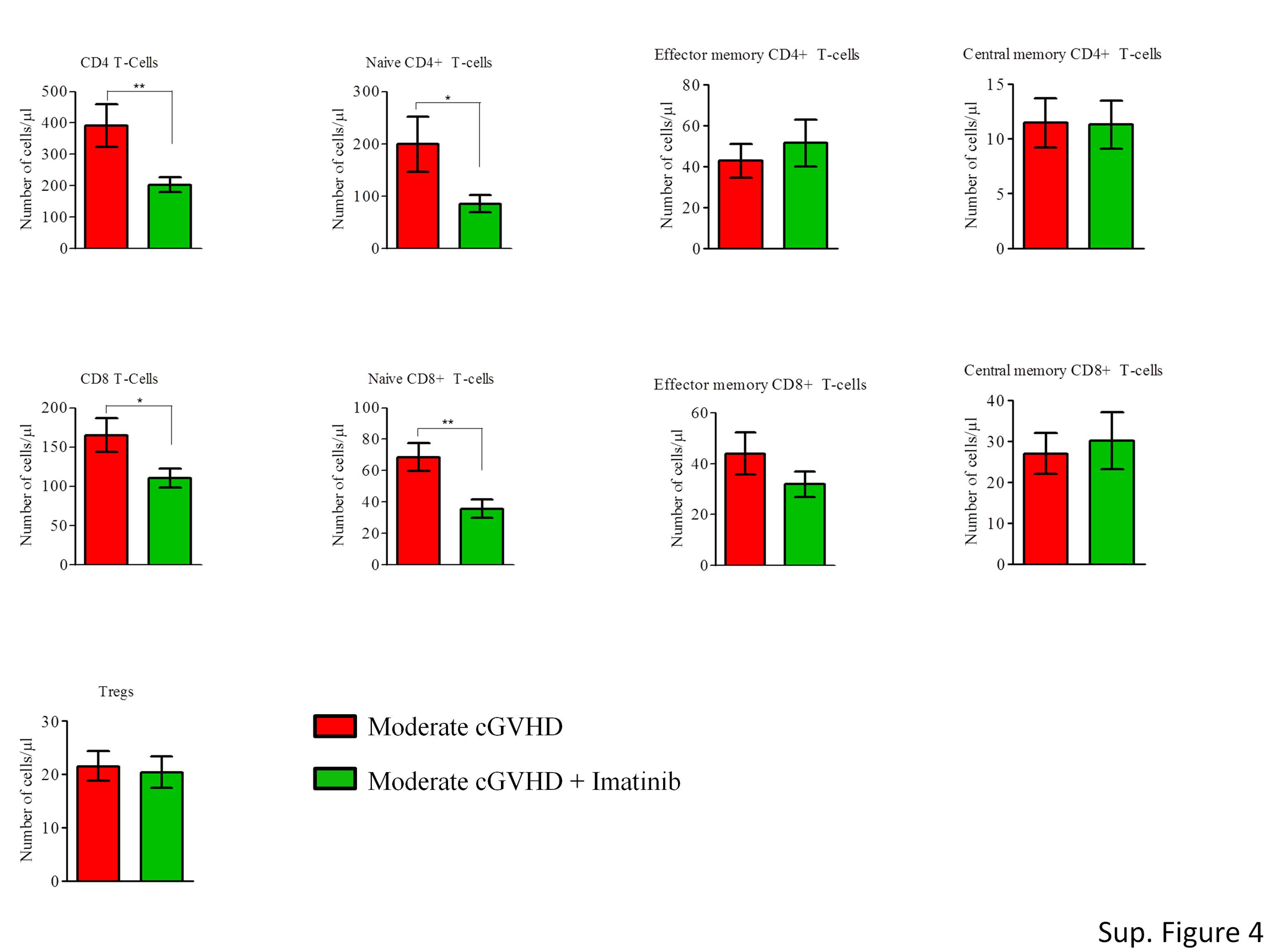

Supplement: S4 Fig — Results indicate that, in a moderate model of scl-cGVHD, imatinib decreased absolute numbers of total and naïve CD4+ and CD8+ T-cells. *P<0.05; **P<0.001. Control: n = 7, imatinib: n = 5. Results are expressed in mean with SEM, Mann-Whitney test. (TIF) [file pone.0167997.s004.tif]

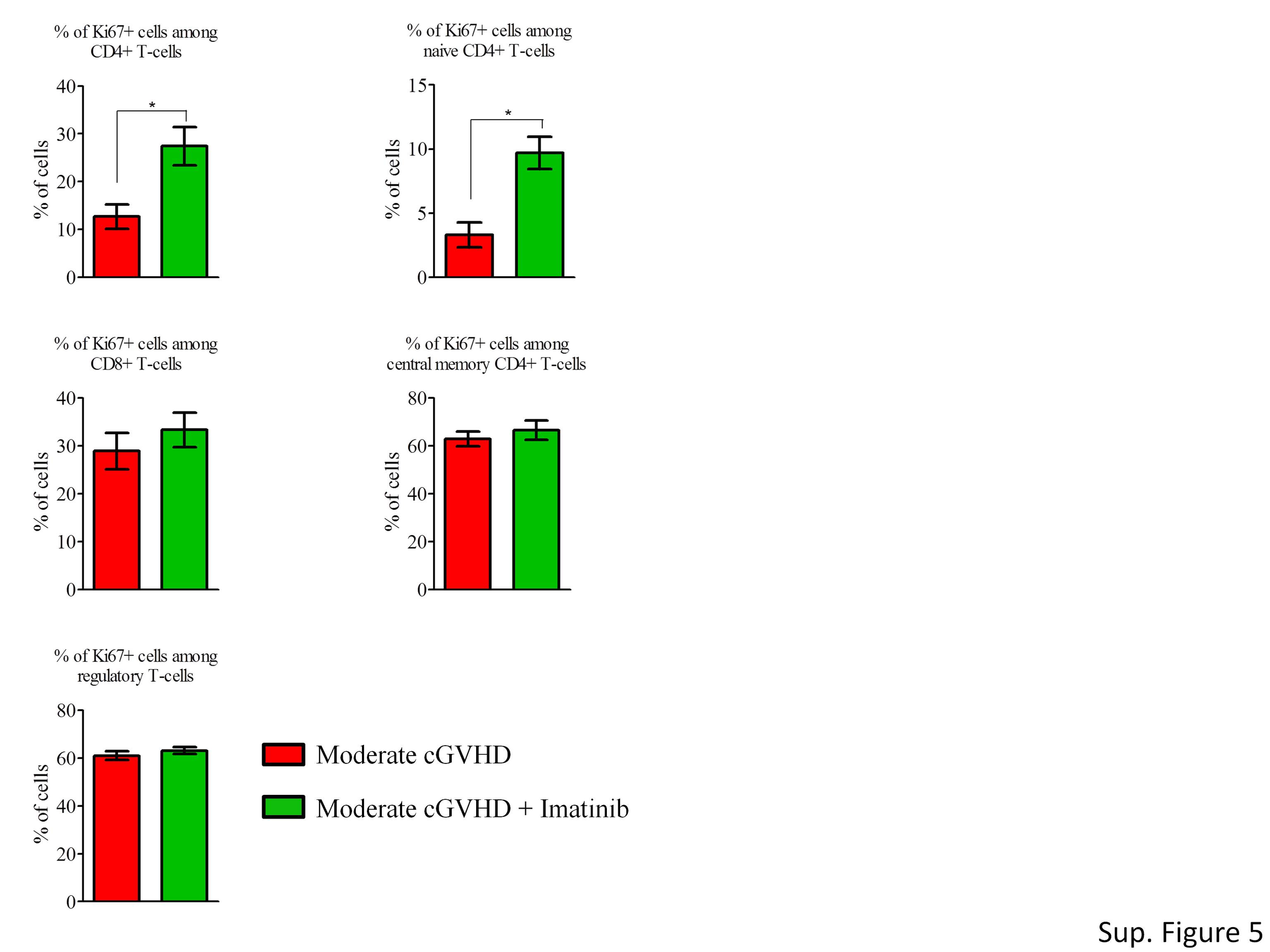

Supplement: S5 Fig — Blood samples were collected at day +35 post-transplant to assess T-cell (subpopulation) proliferation. The results indicate that the expression of KI67 by total and naïve CD4+ T cells was higher in imatinib-treated mice (n = 5) than in controls (n = 7). *P<0.05. Results are expressed in mean with SEM, Mann-Whitney test. (TIF) [file pone.0167997.s005.tif]
